# The Outsider Within. Anticolonial Critiques of Humanity and the Cosmopolitan Vision

**DOI:** 10.1111/1468-4446.13188

**Published:** 2025-01-20

**Authors:** Daniel Davison‐Vecchione, Filipe Carreira da Silva

**Affiliations:** ^1^ Independent Scholar Leatherhead Surrey UK; ^2^ Institute of Social Sciences University of Lisbon Lisbon Portugal; ^3^ Selwyn College University of Cambridge Cambridge UK

**Keywords:** cosmopolitanism, Georg Simmel, humanism, Leopold Senghor, Patricia Hill Collins, stranger

## Abstract

This article re‐examines the anticolonial critique of the concept of ‘humanity’. It uses the example of Leopold Senghor to show the extent to which this critique is shaped by their sociological marginality. Drawing on Georg Simmel's discussion of the ‘stranger’ and Patricia Hill Collins's discussion of the ‘outsider within’, the study rethinks the production of knowledge in racially structured societies. As ‘outsiders within’ colonial empires, anticolonial thinkers from the 1930s to the 1960s challenge the idea of a universal humanity used to justify colonialism and expose its racial stratification. Their critique helps to end colonial domination and develop a more robust conception of common humanity, aligned with a genuine cosmopolitanism that resists exploitative manipulation and promotes anti‐racist agendas. By exploring the critical potential of the figure of the stranger or outsider within, this study invites sociologists to integrate diverse perspectives into sociological discourse and to promote a cosmopolitan epistemology that combines particular and universal insights.

## 1 Introduction

This article builds on Ali Meghji's insight that ‘as long as sociology ignores anticolonial sociology, it remains a fundamentally anti‐sociological discipline’ (2023, 209). As climate activists, AI critics, and those nostalgic for past eras invoke ‘humanity’ to advance their agendas, it is crucial to question what—and who—this concept includes. While these appeals often suggest shared moral solidarity, they risk concealing issues of inclusivity and authority. In this light, the mid‐twentieth‐century anticolonial critique of ‘humanity’ becomes relevant, urging us to consider whose voices and experiences are represented. As Julian Go notes, anticolonial thinkers like Frantz Fanon and Mabel Dove offer ‘critical alternative’ sociological insights by representing marginalized perspectives striving for recognition and worth (Go 2023, 291).

We revisit the anticolonial critique through the concept of the ‘outsider within’. In her 1986 article on Black feminist thought, Patricia Hill Collins describes Black women in academia as ‘outsiders within’. Despite their participation, these women remain ‘outsiders’, similar to the historical role of African American women as ‘privy to some of the most intimate secrets of white society’ (Collins 1986, S14). Collins argues that bringing these perspectives into sociology can reveal realities hidden by orthodox approaches (Collins 1986, S15). In particular, Collins suggests that, by embracing their outsider within status, Black feminists move their disciplines towards a humanist vision of freedom and solidarity (1952/1986, S21). The outsider within bridges the gap between particular knowledge from specific social positions and a universal perspective on the human condition, linking to broader debates about standpoint theories. Crucially for our purposes here, Collins (1952/1986, S15) refers to Georg Simmel's concept of the ‘stranger’, who he defines as a figure who is simultaneously ‘near and far’ from the group (1908/2009, 604).^1^ Simmel's stranger, related to a cosmopolitan personality, perceives the universal in the particular and vice versa (Marotta 2012, 681).

Despite Collins's reference, existing scholarship rarely explores the connections between Simmel's stranger and the concept of the ‘outsider within’. One contributing factor seems to be that the introduction of this concept provided a fundamental legitimising function for Black feminist and intersectional perspectives, which in 1986 had yet to become mainstream in sociology, with the Simmel reference simply taken to serve this legitimising function. This is unfortunate. By reducing the Simmel reference to a mere instrument of legitimisation, we overlook what Collins may have valued in Simmel's work. Even if she drew on Simmel for his authority in the field, she probably saw an intellectual connection between her concept of the ‘outsider within’ and Simmel's idea of the stranger. Moreover, Simmel's exploration of the stranger offers deep insights into social ontology and epistemology that could add valuable perspectives on issues that the ‘outsider within’ concept touches on but does not fully explore.

This article links Simmel's concept of the stranger with Collins's outsider within to make both conceptual and historical contributions. Conceptually, it draws on Simmel to examine knowledge production in structurally racist societies and proposes a ‘cosmopolitan epistemology’. Cosmopolitanism suggests a global community in which we respect and value diverse ways of life. As Kwame Anthony Appiah puts it, it is ‘universality plus difference’ (2007, 151). A cosmopolitan epistemology seeks to include different perspectives without treating them as isolated silos of knowledge, thus acknowledging different viewpoints and their insights while avoiding the limitations of strong standpoint epistemologies. In this respect, our project has some resonance with the efforts of scholars such as Faye Harrison to refine the outsider within in the service of a decolonial anthropology that allows ‘a diversity of anthropologists—diverse in intellectual perspectives as well as sociocultural, geographic, and national origins—to meet and productively engage each other at the “crossroads of knowledge”’ (2008, 4).

To develop this cosmopolitan epistemology, we revisit the anticolonial critique of the 1930s–1960s, when Black thinkers, as ‘outsiders within’ colonial structures, bridged empire and decolonisation. Figures such as Frantz Fanon and Leopold Senghor challenged the racialised hierarchy embedded in colonialism and advocated a universal humanism aligned with anticolonial goals. Aided by a particular form of stranger‐relation to their ‘host’ society, these thinkers constructed a critical cosmopolitanism that redefined humanity in an inclusive way. This approach underpins analyses of race, ethnicity and knowledge, and promotes a universal humanism that opposes oppression without resorting to particularism, thereby enhancing both self‐awareness and social critique.

## 2 Collins's Outsider Within: Knowledge, Critique, and Strangerhood

We will consider Simmel's own thoughts about the stranger in the next section: here we are interested in what Collins sees as significant about the stranger's ‘near yet far’ position for her theorisation of the outsider within. Collins identifies three benefits of occupying this position: (i) what Simmel terms the ‘objectivity’ that arises from a specific combination of closeness and remoteness and of concern and indifference; (ii) how others in the group may be more inclined to treat the stranger as a confidant because the stranger is bound by fewer of the group's constraints and has more fluid relations with the other group members; and (iii) how the stranger's near‐yet‐far position might allow them to recognise patterns that are more difficult for those immersed in the group to notice (Collins 1986, S21; Simmel 1908/2009, 602, 603).

Collins connects her ideas to Thomas Kuhn's concept of a paradigm, defined as the shared constellation of beliefs, values, and techniques within a community (1962, 175). She emphasizes that knowledge systems are never complete but serve as frameworks for ‘thinking as usual’ (1986, S25), where facts gain meaning through their theoretical contexts, and theories make sense of observations by framing them as facts. Collins identifies three dimensions to a Kuhnian paradigm community: shared worldviews, the process of becoming an insider, and the maintenance of insider status (1986, S25, S26). She notes that Black women may experience a poor fit between sociological paradigms and their own experiences, positioning them as ‘strangers’ who, unlike ‘normal’ sociologists, are more likely to notice and challenge assumptions and anomalies within the discipline (1986, S26, S27). Collins observes that Black women scholars often encounter two key problems: the exclusion of facts about Black women in sociological paradigms and the distortion of those that are included (1986, S27, S28). Black feminist scholarship addresses core sociological questions, such as which social elements are worthy of study and how well current concepts, such as work and family, capture lived realities. Black women who critically engage with sociological paradigms, while grounded in their own experiences, can offer unique insights that not only enrich the study of Black women, but also address fundamental questions in sociology. Collins rightly notes that Black women are not the only outsiders within sociology: Black men, working‐class individuals, and anyone who, despite sharing the benefits of white male insiderism, has ‘never felt comfortable with its taken‐for‐granted assumptions’ can also potentially institutionalise outsider within ways of seeing in the discipline as a whole. This ‘creative tension’ between personal experience and sociological critique encourages new perspectives on the anomalies inherent in the discipline (Collins 1986, S29).

Collins links the outsider within to issues of marginality and power, challenging views that see outsiders as either ‘grateful ambassadors or unwelcome intruders’ (1998, 24). She highlights how marginal positions can foster intellectual and ethical strength, seeing them as sites for ‘oppositional perspectives’ (1998, 24). For those in these positions, such as Black women, knowledge about dominant groups can be gained without full access to their power. This framework is consistent with intersectional analysis, which shows how overlapping systems of race, class, gender and other oppressions shape unique insights. From these ‘borderland spaces’ (Collins 2019, 32), outsiders can reveal power dynamics and promote opportunities for democratic exchange.

In discussing African‐American intellectuals such as Sojourner Truth, Collins emphasises the role of migration and mobility in shaping the knowledge they produced. These thinkers did not remain within a centre of power, but instead ‘breached group boundaries’, challenging norms and exposing ‘segregated spaces’ (Collins 1998, 258, 259). While Collins notes that outsider‐within perspectives are not inherently progressive, this position allows Black feminist thought to be both ‘particular and universal’ (Collins 1998, 266). Black feminist thought, as an intellectual struggle for freedom, engages with other movements and balances its unique context with the broader coalition‐building efforts essential to social justice.

Collins later identified two key issues in the evolving use of ‘outsider‐within’. First, she noted that the term had shifted from describing a group's historical context to a more individualised identity, potentially distracting from the social hierarchies that create these outsider‐within positions (Collins 1999, 85, 86). She feared that this shift might dilute the specific experiences of Black women in the U.S. Second, Collins observed that the term had been commodified in academia, where the value of Black women was reduced to their marginal status, which inherently limits real power (Collins 1999, 88). In the following sections, we will draw on Simmel's concept of the stranger and examine Black anti‐colonial thinkers to address these challenges.

## 3 Simmel's Stranger and the Sociology of Knowledge

As it is central to our reconsideration of the conditions of production of knowledge in structurally racist societies, it is worth considering the epistemological implications of Simmel's approach to the stranger and how these relate to Collins's theorisation of the outsider within. Whilst usually read as a standalone piece because of its frequent inclusion in edited collections,^2^ in its originally published form, ‘The Stranger’ is a short excursus within the chapter on ‘Space and the Spatial Ordering of Society’ in Simmel's 1908 book *Sociology* (Simmel 1908/2009, 543–620). Here Simmel examines how major aspects of space are formed socially, how historical changes make different types of spatial relationship possible, and how spatial context and the use of space help create and maintain forms of human sociation. In Simmel's (1908/2009, 601) view, ‘every relation among people’ contains a ‘union of the near and the far’ and this is embodied in the social type of the stranger: a figure who is neither entirely inside nor entirely outside the host group. The stranger in this sense is not a wanderer, but rather a potential wanderer: not ‘one who arrives today and leaves tomorrow’, but rather ‘one who comes today and stays tomorrow’ (Simmel 1908/2009, 601). As Simmel (1908/2009, 601) goes on to say, ‘[t]he stranger is fixed within a certain spatial area—or one whose delimitation is analogous to being spatially limited—but the position of the stranger is thereby essentially determined by not belonging in it from the outset, and by introducing qualities that do not and cannot originate from the stranger’. Therefore, the stranger embodies the paradoxes of distance and proximity: a figure who is ‘near and far *at the same time*’ (Simmel 1908/2009, 604). The stranger is part of the group, and the rest of the group often relies on the stranger. Yet they are connected through only the most general commonalities.

From this, one can see why Collins considered Simmel's stranger a helpful starting point. Like the stranger, the outsider within is someone who embodies the paradoxical unity of social nearness and remoteness: a participant in a group who is neither wholly an insider nor wholly an outsider. In Simmel's (1908/2009, 602—our emphasis) view, ‘[b]y not being radically committed to individual components or one‐sided tendencies of the group, the stranger faces all of them with the special attitude of the “objective” person, which does not mean, perhaps, a mere aloofness or disengagement but *a particular form of the far and near*’. Like those African‐American women intellectuals Collins analysed, the Simmelian stranger's mobility enables him to transgress group boundaries and produce specific kinds of knowledge. As indicated previously, Simmel's stranger is closely linked to the idea of the modern, cosmopolitan personality type who can perceive the universal within the particular and vice versa (Marotta 2012, 681). Echoing the general importance Simmel gives to the ‘third’ as an element in relational configurations, the stranger has an energising and qualitatively transformative effect on the host group, bringing new qualities into the group from the outside, as well as activating hidden potentialities within the group (Pyyhtinen 2009, 122; Levine 1977, 20; Karakayali 2003, 53).

Much of the Anglophone reception of Simmel's stranger has been via the concept of the ‘marginal man’ that Park (1928) and his colleagues in the interwar Chicago School of sociology developed in their studies of urban immigrant communities. Unlike the marginal man, whom Park conceptualised as a racial or cultural hybrid who aspires to but is denied membership in the dominant culture and consequently suffers from restlessness, intensified self‐consciousness, spiritual instability, and malaise, Simmel's stranger does not seek to be assimilated (Levine 1977, 17). Likewise, Simmel does not suggest that the stranger is necessarily an ethnic or racial ‘other’ and numerous historical examples that would fit his definition of a stranger group, such as the metics of Ancient Greece, were not identified in such terms (Karakayali 2003, 47): a metic was simply someone who immigrated to Athens fleeing persecution or poverty in their home country. This is important for our present purposes because, although—unlike Park's interpretation—Collins's reading of Simmel's stranger does not assume an endpoint of assimilation, like Park, Collins still associates the stranger with a racialised and socially marginalised figure. This suggests that, while Collins (1986, S15) uses the outsider within to emphasise the potentially positive features of marginality and strangerhood in deliberate contrast to Park and other established figures in American sociology, her reinterpretation of Simmel's stranger is in several respects still undertaken in the terms originally defined by the Chicago School.

This leads to a further complication: although Simmel identifies the stranger as a particular social type, he also presents strangeness as a general condition of modernity. Indeed, for Simmel, a degree of separation, estrangement and alienation is inscribed in modern human existence itself (R. Cooper 2010). As Goodstein (2017, 315) observes, in Simmel's view, ‘the social is constituted by beings whose being is defined by their not entirely belonging to the whole of which they are the constitutive elements or parts’. Since ‘[s]ociety is defined […] by figurative boundaries, in and through symbolic forms that are also enacted’, to ask Simmel's famous question ‘How is society possible?’ is ‘to ask about the often alienating implications of representation and of our existence as beings who represent ourselves to ourselves, who are others for ourselves’ (Goodstein 2017, 315). Thus, the stranger reflects Simmel's broader commitment to understanding the constructed quality of everyday life (form) and how this shapes experience and, in his later writings, life itself (Featherstone 2021, 206). Under modern conditions, any hope for a non‐representational view of ourselves or of others is irrevocably lost. In a fundamental sociological sense, then, we are always strange (Featherstone 2021, 208; Fitzi 2020, 97).

This raises an important question: how can the outsider within yield unique and beneficial insights by virtue of being a Simmelian stranger when, in Simmel's view, *everyone* in modern society is potentially a stranger? One solution is to think not in categorical or binary terms (stranger/insider), but in terms of *strangerhood*, that is, a continuum of proximity and distance, with the intensity of strangeness depending on, amongst other factors, where one lies on the continuum. This enables strangerhood to encompass (i) social actors who are strangers because of how the host society has categorised them and (ii) social actors who experience a more context‐based sense of strangeness, whilst still allowing one to recognise the differences between these social experiences of strangerhood (Marotta 2009, 275). The concept of the ‘outsider within’ tries to capture the former, occupying the part of the continuum where strangeness is felt more intensely and has a more structural configuration. It is in this sense that anticolonial thinkers occupy a Simmelian stranger position. Conceptualising the outsider within this way avoids the kind of radical decontextualisation of the outsider within that Collins was wary of, highlighting the structural contexts that produce outsider‐within locations, while still acknowledging the outsider within's connections to other forms of strangerhood.

This leads to the epistemological question of the ‘objectivity’ of the stranger. In contrast to the positivist view, which sees subjectivity as an obstacle to knowledge, Simmel argues that true objectivity involves the subjective (Marotta 2012, 685). In his view, historical knowledge relies on bridging the self and the other, as historians, like strangers, share some common ground with their subjects. The historian uses personal identity to understand beyond themselves, with unique, personal experiences enriching their understanding of others (Simmel 1977, 88). This relational perspective suggests that objectivity depends on the distance from which knowledge is observed and understood (Fu 2022, 601), with the objectivity of the stranger arising from their unique, reciprocal relationship within social contexts, forming ‘a particular mode of being‐related to the dominant perspective, […] embedded in a social relation or configuration that includes *both* perspectives’ (Goodstein 2017, 310).

Finally, it is worth considering the connection between Simmelian strangerhood and cosmopolitanism in light of Simmel's analysis of the modern metropolis. As he puts it in his famous 1903 essay ‘The Metropolis and Mental Life’, by ‘transcending [the] purely tangible extensiveness’ of ‘the immediate size of the area and population’, the metropolis ‘becomes the seat of cosmopolitanism’—‘in contrast with the trivialities and prejudices which bind the small town person’, the metropolitan citizen is free ‘in an intellectualized and refined sense’ (Simmel 1971, 334). In Thomas Kemple's (2014, 278) words, Simmel sees the metropolitan type ‘as both a member of a *globalizing society of strangers* and as *the local seat of cosmopolitan intellectuality*’.

All this provides some initial clues as to how anticolonial thinkers like Senghor used their positions as outsiders within to lay the groundwork for a critical‐cosmopolitan epistemology. By occupying a socio‐spatial position marked by both inclusion and exclusion, such writers had a specific mode of being related to the dominant perspective, embedded in a social relationship or configuration from which they could incorporate elements of the dominant perspective while maintaining a critical distance from it. Since their strangeness was partly the result of how they were categorised by the host society, it was not the general existential condition of modern people, but a particularly intense personal experience. The ‘objectivity’ of these thinkers is relational, blending objectivism and subjectivism in a way that takes into account, on the one hand, the thinker's particular personality and experience and, on the other, the thinker's more general commonalities with the members of the host or dominant group. Finally, as the following section will explore in detail, from their position as strangers in the modern metropolis of Paris, they were able to transcend a purely tangible sense of extensiveness and cultivate a truly cosmopolitan sense of connection between the local and the global. Since the prevailing colonialist discourses only partially included Black colonial subjects in the concept of ‘humanity’, these outsiders were able to use their specific stranger position within the modern, colonial metropolis to critique and reconstruct humanism.

## 4 Strangers in Paris: Black Anticolonial Thought and the Critique of Common Humanity

In the 1930s, Paris was a global hub, attracting people from all over the world to work and study. In particular, the city attracted a large community of students and thinkers from the colonies—twentieth‐century metics, so to speak. In this modern, cosmopolitan metropolis, they experienced both the alienating (Vaillant 1990, 127, 128) and liberating effects described by Simmel. As with those African‐American intellectuals Collins examined as outsiders within US society, the experience of migration and mobility shaped the knowledge they produced and allowed them to challenge norms. Soon, Paris became a hotbed of anticolonial thought. Various groups quickly emerged, including that around Nancy Cunard, whose monumental *Negro* (Cunard 1934) brought together an international constellation of anti‐racist and anticolonial thinkers and artists, and the group of contributors to *La Revue du Monde Noir*, which included some of the most important Black writers of the following decades, notably Louis Achille, René Maran and Félix Eboué. It is around this journal, published for six months in 1931, that the Negritude movement begins to take shape out of the experience of strangeness in the colonial metropolis. Looming large over this community of outsiders within the French Empire is the towering figure of an African‐American thinker: W.E.B. Du Bois, whose Pan‐Africanism and concept of double consciousness provided important inspiration (e.g., Du Bois 1934).

In what follows, we focus on the writings of an emblematic Black anticolonial outsider within: Leopold Sedar Senghor (1906–2001), co‐founder of the Negritude movement with Aimé Césaire (1913–2008). The two men met in Paris in September 1931 and quickly became lifelong friends (Vaillant 1990, 89). Intellectually, however, their understanding of Negritude developed in different directions: for Césaire, Negritude was primarily an attitude; for Senghor, it was an objective reality. In a recent important article on the Negritudinists and the ‘crisis of man’ in the mid‐twentieth century (2018, 775), Gili Kliger reminds us that C. L. R. James once said of Césaire, ‘He was able to make this ferocious attack upon Western civilization because he knew it inside out’ (cited in Kliger 2018, 779). Kliger zooms in on Césaire's *Discourse on Colonialism* (1950) to dissect the critique of humanism from the perspective of a Black anticolonial stranger; here we undertake a similar task by reference to Senghor's literary and political writings. Specifically, we ask: To what extent did Senghor's social position as an outsider within the French colonial empire inform his social critique of humanism and colonialism?

For Senghor, the experience of being an outsider within is the fertile soil in which the seeds of negritude will be planted (Bâ 1973; Markovitz 1969). It is a search for self‐discovery—Who am I, asks Senghor? *Not* a white Frenchman (Senghor 1964a, 405, 406). From this singular position Senghor will ‘begin again’ as a ‘new Negro’,^3^ a deeply disturbing personal experience. It is also a collective experience shared by other students from all over the empire who drew together out of a shared sense of strangeness—a perception that, at least temporarily, obscured the differences amongst them. In Senghor's case, this personal traumatic experience will eventually translate into a sophisticated critical re‐examination of Western humanism: a re‐examination that will lead him to critically rethink what ‘the human’ is, what can sustain a shared sense of humanity, and the very contours of humanism as a philosophical doctrine. Politically, this critical engagement with humanism is aimed at keeping the best of French civilisation whilst planting the seeds for African independence. This critical re‐examination will be undertaken from the perspective of what Senghor calls his ‘Negritude’, that is, ‘the ensemble of cultural values of the Black world, including the Americas’ (Senghor 1964a, 269). Du Bois's *The Souls of Black Folk* (1903), with its master trope of double consciousness, describes a similar experience of twoness. Unsurprisingly, Senghor will later call Du Bois a father of Negritude and firmly believes in the existence of an essential, shared Black experience in and beyond the US.

Behind Senghor's understanding of Negritude are two intellectual developments that characterise the postwar French intellectual scene. The first is the rediscovery of the ‘young Marx’ of the *Paris Manuscripts*. Like for so many others at the time, Senghor's encounter with the young Marx in the deputies' library of the Palais Bourbon after the war signals a much‐needed recentring of socialism on the problematic of alienation (Senghor 1948). That this humanist concern with alienation cannot be easily separated from the unique material form of Marx's early manuscripts has been the object of renewed attention recently (Silva and Vieira 2019, 62–96). The second is the work of de Chardin (1961), whose concept of *plus‐être* (a situation not just of well‐being but of greater being, in which all the potential of human reason and emotion is realised) will help Senghor revisit the problem of alienation through the figure of the humanist, as one who wishes to make ‘man’ more truly human, ‘by making him participate in everything that can enrich him in nature and history’ (Senghor 1962, 22, 25). This translates into what Senghor calls, following Chardin, a civilisation of the universal (Senghor 1977). Whilst dehumanised Europe will benefit from Africa's gift of greater emotional and spiritual development, so Africa, currently paralysed by colonial extractivism and capitalist exploitation, will benefit from the development of analytical reason and a more inquisitive spirit. The range of human possibility will historically be finally realised in this higher civilisational form (Senghor 1964b, 134–154; 1962, 15–65).

The synthesis between humanism and socialism reaches its most complete form in the late 1950s in two papers Senghor presents at the First and Second Congresses of Black Writers and Artists.^4^ ‘L'esthétique négro‐africaine’, from 1956, is a discussion of African culture and aesthetics. ‘Eléments constitutifs d'une civilisation d'inspiration négro‐africaine’, from 1959, offers an analysis of the facts of the ‘African Negro’ civilisation, which encompass ‘the social institutions, such as the organizations and cultural characteristics, not to say “works”, the “totems” and themes, […] which are found among all Negroes—even among those of America—precisely because they carry with them universally human values’ (Senghor 1964a, 263). Humanism, once reappropriated through the lenses of Negritude, ceases to be a Western creation, which can be deployed to justify human alienation and unfreedom, and becomes a truly universal category (Senghor 1965). Crucially, this movement towards universality is brought about by strangeness as only the experience of being an outsider within gives one the deep familiarity of a reality that one wishes to build anew.

Senghor's new humanism has notable limitations. Critics (Towa 1971; Adotevi 1972, Soyinka 1976) argue that Negritude can be seen as a conservative, melioristic response to colonialism. Senghor's reliance on Catholic social doctrine overlooks Christianity's role in colonialism. His adherence to socialism in a mostly post‐socialist world raises questions, as does his essentialist view of a unified ‘Black African’ civilization. Additionally, Senghor's establishment of a repressive one‐party regime in Senegal (1960–1980) challenge his writings on freedom and opposition, despite his eventual peaceful stepping‐down (Bobin 2020). This issue resembles Collins's view on the limitations of outsider‐within status, where permanent marginality rarely leads to real power. Did Senghor's shift from intellectual to statesman diminish his outsider perspective, complicating the role of such figures in political movements when they gain power?

Whilst these concerns are well‐grounded, we maintain that there is considerable value in Senghor's new humanism providing one approaches it with a critical attitude. Here it is worth bearing in mind that more recent literature has disputed the foregoing interpretation of Negritude, pointing out its radical attempt at transforming a historical relation of domination into one of reciprocity (Kliger 2018, 799). Our own re‐examination of this historical episode comes closer to this more recent scholarship; in the next and final section, we delineate a cosmopolitan model of knowledge‐production based upon it.

The new humanism delineated by Senghor is a complex and rich project, encompassing several dimensions. First, Senghor rejects anthropocentric humanism, the ultimately self‐centred perspective he associates with Western modernity and whiteness (Vaillant 1990, 257). Negritude, which emerges out of an outsider within experience, offers an alternative kind of humanism based upon a cosmological unity between the human species and the natural environment inspired by the teachings of what he terms ‘Negro‐African’ civilisation. From this animist perspective, all objects in the social and natural worlds share a common essence: they are all part of the biosphere. All beings, human and nonhuman, as well as all previous and future generations, are linked in a communion of living and dead, spirit and flesh. Senghor writes: ‘Negro‐African animism makes the Earth, the principal means of production among peasant peoples, into a person, a spirit. […] Not only is the Earth a person, but so is the wealth which it bears or contains, the mines, the waters, the animals and the trees’ (1964a, 282). This is not to say that anticolonial outsiders within necessarily reject anthropocentrism in the name of a cosmological vision like Senghor's. Amílcar Cabral's rejection of anthropocentrism, for example, stems from his background as a soil scientist and has nothing to do with animism. But it does mean that anticolonial outsiders within tend to ask the same question—is ‘Man’ really the centre of all things and the ultimate apex of human civilisation, of which colonialism is but a necessary and temporary step?

Second, Senghor conceives of the human‐nonhuman boundary as a political construction that has worked for the benefit of the few at the expense of the many, colonised peoples and nature included. This is because, Senghor argues, we have forgotten that Man is an animal with special characteristics, which places Man both within nature and outside it. ‘The second characteristic of this ontology is the eminent place that living man, the Being, occupies in the hierarchy of the forces’, Senghor writes. Man occupies a central place alongside ‘the cosmic forces, the stars and inferior forces, embodied in animals, plants, and minerals’ that make up the universe; in this sense, the human species exists within nature. Yet this central position in the universe renders Man ‘more alive and real’ as a ‘free being, the freest being possible. As such, freedom, which transcends the contingent factors, is at the heart of the problem; it is the umbilical knot of the world’ (Senghor 1964a, 275). The new humanism is built upon this often forgotten yet eternal truth.

In these important respects, Senghor echoes Simmel. Simmel's sociology emphasises relationality, hence his understanding of strangerhood as a relational position in social space. Importantly, Simmel does not limit his sociology to relations between humans. As can be seen in such works as his detailed study of money and his essay on the bridge and the door, Simmel (1900/2011, 1994) asks how objects establish connections and boundaries. Similarly, as David Beer (2019) explains, Simmel's later writings, especially those collected in *The View of Life* (Simmel 2010), focus on the relationships between worlds, lives, and fragments. Throughout his work, Simmel returns to the question of the human and its relationship to the nonhuman. As Mark Featherstone (2021, 217) suggests, Simmel offers a ‘non‐human humanism’: a humanism ‘organised around a recognition that there is nothing special about human beings in the scheme of life in itself, that we are limited, vulnerable, and only survive on the basis of our necessary immersion in a socio‐eco‐system defined by relationality and openness to other forms of life’. The same could be said of Senghor's attempt to build a non‐anthropocentric humanism and his conception of the human‐nonhuman boundary as a political construction that obscures how human beings are both inside and outside of nature.

Third, Senghor makes an influential critique of cultural domination by European colonial powers. His main argument is deceptively simple. Only by ‘assimilating, not [by being] assimilated’, can colonised peoples become truly free and independent. ‘Writers and Artists must play, and are playing, a leading role in the struggle for decolonization’, Senghor notes. He then reminds his reader that ‘politics, the administration of the Polity, is only one aspect of culture, which, starting from cultural colonialism in the form of assimilation, is the worst of all’ (1964a, 293). Senghor's rejection of assimilationism, a key component of French colonialism and a topic of analysis of French colonial sociology at the time (Steinmetz 2017, 641, 642), is directly derived from his personal experience of being a stranger in 1930s Paris. Yet it is this same experience that leads him to emphasise the agency of the oppressed: assimilation (of values, cultures, knowledge) is to be kept as long as it works, following Negritude, for the benefit of all. In this respect, Negritude stands in stark contrast to the civilisational tabula rasa approach that the Khmer Rouge would apply to Cambodian society and culture in the mid‐1970s.

Fourth, a civilisation of the universal nurtures not only instrumental rationality, but also communicative reason. Europe's growing reliance upon the former has isolated Europeans from nature and led them to see it as alien. This European attitude views nature as valuable only insofar as it can be made useful to ‘man’—a purely instrumental attitude that Senghor contrasts with that of the Black African: ‘Aroused by powerful determination, he kills the Other and, in a centripetal movement, he finds means by which to use it for practical purposes. He assimilates it. Such is the European white man […] “I think, therefore I exist,” wrote Descartes. The African Negro could say: “I feel the Other; I dance the Other, therefore I exist”’ (1977, 237). This emphasis on embodied forms of being, thinking and knowing (Ringmar 2023, 25–109) provides an alternative epistemology to the despotism of reason associated with traditional Western humanism.

Fifth, this critique of instrumental reason underpins a more specific critique of alienated labour. This is based upon two premises. On the one hand, Senghor stresses the development of *Homo sapiens*, thinker and artist, from Homo faber, the producer and the worker. This signals the basic importance of free meaningful labour, as opposed to forced or exploitative work. Equally important, as the African cultural tradition shows, is to acknowledge the porosity between instrumental and communicative reason in concrete contexts of action (Vaillant 1990, 257). As Senghor stresses, by singing whilst working, one can have one foot inside the labour market whilst keeping the other outside it. It is worth citing the full passage:

And the labour itself is not alienated. It is not an imposition, but a source of joy. Because it is free, it permits the intensification and realization of being. In Negro‐African society; it will be noted, work on the land is the most noble. That is because this work enables man to be in tune with the universe it is performed in the rhythm of cosmic forces. And the Negro‐African, feeling himself to be in unison with the universe, works in rhythm, accompanied by musical instruments. Working songs by the thousand—songs of the peasant, the herdsman, the fisherman, the craftsman—songs which *accomplish* the work. Negro labour, Negro rhythm, Negro joy which is liberated by work and therefore is liberated from work!

(1964a, 284)

Sixth, there is an important ethical dimension in Senghor's new humanism. This is closely related to the role religious values play in his thought, and in Negritude more generally. Born into a Catholic family, educated by Catholic missionaries, and influenced by Catholic ‘spiritualists’ such as Jacques Maritain and Teilhard de Chardin (Senghor 1962), Senghor rejects materialist accounts of human existence and instead defines ethics as ‘active wisdom. It consists, for the living man’, he observes, ‘in recognizing the unity of the world and in working towards its ordering’ (1964a, 285). Hence understood, active wisdom is directly related to strangeness. This is because of the human species' singular relation to nature's contingent facts. Following the humanist Marx, who ‘asserted this truth with force in a posthumous work which is surprisingly unfamiliar’, Senghor argues that only ‘man can dream and express his dream in works which transcend it. And in this field the Negro is king’ (1964a, 290). This transcendence is at the heart of Senghor's socialist humanism. ‘This is the very meaning of the *dialectic* movement in history, at the culmination of which man's liberty springs forth’, he writes. ‘Thus, one sees that the use to which we put the socialist method is humanistic. The method serves only as framework. It is our duty, particularly, to discover the mediations and define their functions. In a word, it is a question of explaining *man by man*’ (1964a, 263). Or, as he puts it later in the essay: ‘The African Negro is thus enclosed in a narrow network of vertical and horizontal solidarity, which hind and sustain him at the same time. This is the most precise illustration of this truth, upheld today by Socialism, according to which *Man lives and is realized only by and in society*’ (1964a, 277).

Seventh, Senghor's new humanism has an important political dimension that is firmly linked to decolonisation. However, as Senghor recognised, the decolonisation of entire nations and cultures is a complex task, complicated by issues such as neo‐colonialism and the persistence of colonial structures. Senghor's approach—socialist humanism—draws on Maritain's *Integral Humanism*, offering a faith‐based alternative to both Soviet communism and Western capitalism, which he saw as politically limiting (1936/1968, 88; see also Thompson 1957, 143). Senghor emphasises the need to centre ‘Man’ to prevent the state or the market from dehumanising individuals, noting that ‘a modern state is not built for the mere pleasure of building’ and must avoid ‘a will to power which deifies the state and crushes Man under the State’ (1964a, 293). Senghor's vision of the postcolony embodies the outsider‐within perspective: *joining* the international community while *rejecting* the dichotomy of Western capitalism versus Soviet communism. Along with Césaire, Senghor advocated constitutional change to transform imperial France into a democratic federation in which the former colonies would have autonomy (Wilder 2015). This federalist approach offered a cosmopolitan alternative that allowed for flexible political alignment, although it was never fully realised (F. Cooper 2014). The contrast with Fanon is instructive. Fanon criticised Senghor's federalist vision as overly conciliatory towards colonial power, dismissing it as ‘pharisaical’ in *The Wretched of the Earth*: ‘And now the moment has come to denounce certain pharisees. Humanity, some say, has got past the stage of nationalist claims. The time has come to build larger political unions, and consequently the old‐fashioned nationalists should correct their mistakes’. He goes on to say that he believes that, on the contrary, ‘[t]he mistake, heavy with consequences, would be to miss out on the national stage. If culture is the expression of the national consciousness, I shall have no hesitation in saying, in the case in point, that national consciousness is the highest form of culture’ (1963/2004, 179). For Fanon, nationalism was essential to building a true internationalism, arguing that ‘national consciousness, which is not nationalism, is alone capable of giving us an international dimension’ and that nationalism, by expressing ‘the will of the people’, could lead to universal values (2004, 179, 180). In short, while Senghor's cosmopolitan embrace of federalism points to a political solution that we today might only recognise as a dead end, in itself a useful historical lesson, Fanon's attachment to national forms of identification leads him to bypass the cosmopolitan epistemology we are articulating here. Somewhat ironically given Fanon's criticisms of Senghor's federalist vision, this epistemology strongly resonates with the cosmopolitan impulse that animates the more authentic humanism Fanon envisages at several points. To take one example from the evocative closing passages of *Black Skin, White Masks*: ‘Superiority? Inferiority? Why not the quite simple attempt to touch the other, to feel the other, to explain the other to myself?’ (Fanon 1986, 231).

All of this suggests that considering the outsider within through the historical example of Negritude in its African expressions might not only complement Collins's view of the Black American position, but also provide an alternative to it. As already acknowledged, there are serious problems with Negritude's positing of a unified, global Black experience or consciousness, including its essentialist implication. However, if we understand its project as one of clarifying the contributions of Black‐majority populations to other societies, undertaken in response to colonialism, which denied such a possibility of contribution, then Negritude seems more like an attempt to critique and rescue humanist universalism than a retreat into particularism. In this respect, Negritude may counterintuitively be more cosmopolitan than intersectionality, because the latter emerges from the experience of minority groups and aims to restore social justice in that particular society, even if social justice can be sought globally.

Thus, revisiting Negritude through the lens of Simmel's stranger and Collins's outsider within provides a different route for scholars to bring sociology closer to the kind of ‘humanist vision’ that Collins saw as implicit in Black feminist work: a vision based on ‘the freedom both to be different and part of the solidarity of humanity’ (1986, S30). While doing so means expanding the outsider within beyond an expressly feminist perspective, by acknowledging the complexities within stranger‐relations, including how one can move between different forms and degrees of strangerhood across different socially structured contexts, the concept can still serve its original, feminist purpose. Indeed, it might even help rectify the comparative under‐analysis of issues of gender and feminism within the writings of influential anticolonial thinkers like Senghor.

## 5 Conclusion

To bring our argument to a close, we return to the question raised at the start: whose voices and experiences are represented when appeals to ‘humanity’ are made, and what might be overlooked? In colonialist discourses, the colonised were not fully included in the concept of humanity. Revisiting Collins's concept of the outsider within through Simmel's ‘Excursus on the Stranger’ sheds light on how anticolonial thinkers like Senghor navigated and redefined their roles within colonial structures. To recontextualise Simmel's words, one could say that Senghor imagined a world in which cities like Paris and Dakar, or in the case of the British Empire, London and Hong Kong, could simultaneously possess ‘the utmost individuality and distinctiveness’ and ‘the elevation beyond all limitation and happenstance of an individually fixed existence’ (Simmel 1908/2009, 564). Senghor's cosmopolitan humanism aimed to dismantle colonial dominance while preserving the broader networks of cultural and economic exchange created by the empire. This perspective invites us to see the outsider within not merely as marginal but as a critical vantage point—a distinct form of strangerhood shaped by colonialism, where the local and supra‐local intersect, challenging fixed notions of belonging and identity.

Senghor's vision reflects a cosmopolitan outlook shaped by his experience as an outsider within the French colonial empire. This perspective allows one to perceive the universal in the particular and vice versa, always involving negotiation and compromise. In terms of the sociology of knowledge, this perspective fosters forms of social self‐knowledge that explore the critical role of the stranger or outsider within—a balancing act between belonging and outsidedness that is activated in real‐world situations. It reconfigures standpoint epistemology to accommodate the outsider‐within perspective, moving beyond a fixed subjective standpoint to demand an open‐ended engagement with the viewpoints of others. This means that particular perspectives are not closed domains exclusive to insiders, but platforms where specific aspects of the universal can be revealed. Cosmopolitan epistemology thus aims to integrate diverse particular experiences into a coherent sociological framework, guided by critique and comparison rather than accepting any perspective at face value. By engaging more explicitly with the work of Senghor and Cabral, sociologists today have two concrete examples of how to negotiate belonging in ways that escape an easy and dichotomous narrative of acceptance versus resistance. Sociologists, too, must assume the role of strangers, simultaneously inside and outside the social worlds they study. This article encourages sociologists to draw on the insights of anticolonial thinkers such as Senghor, Césaire, Fanon and Cabral, who embody this approach by combining specific cultural points of view with universal human concerns.

## Data Availability

Data sharing is not applicable to this article as no new data were created or analysed in this study.

## Endnotes

^1^
Within the full‐length English‐language publication of *Sociology* cited here and throughout this article (Simmel 1908/2009), the ‘Excursus on the Stranger’ is at 601–605.

^2^
See for example, Simmel (1971, 143–149), Simmel (1950, 402–408), and Park and Burgess (1921, 322–327).

^3^
‘I accepted myself such as I was (…) I was born again’ (Senghor in Dorsemaine, Fierro, and Masson 1978, 83, 84).

^4^
Organized by Alioune and Christiane Diop of *Présence Africaine*, these Congresses brought together dozens of prominent Black writers and artists from the English‐ and French‐speaking worlds, thus confirming the ‘basic premise of Negritude, namely that all Black people had much in common. Its importance was underlined by the fact that the meeting was held at the Sorbonne, the centre of French humanistic learning’ (Vaillant 1990, 284–285).
